# Molecular diagnostics for genotypic detection of antibiotic resistance: current landscape and future directions

**DOI:** 10.1093/jacamr/dlad018

**Published:** 2023-02-17

**Authors:** Ritu Banerjee, Robin Patel

**Affiliations:** Division of Pediatric Infectious Diseases, Vanderbilt University, Nashville, TN 37232, USA; Division of Clinical Microbiology, Department of Laboratory Medicine and Pathology Mayo Clinic, Rochester, MN, USA; Division of Public Health, Infectious Diseases, and Occupational Medicine, Department of Medicine, Mayo Clinic, Rochester, MN, USA

## Abstract

Antimicrobial resistance (AMR) among bacteria is an escalating public health emergency that has worsened during the COVID-19 pandemic. When making antibiotic treatment decisions, clinicians rely heavily on determination of antibiotic susceptibility or resistance by the microbiology laboratory, but conventional methods often take several days to identify AMR. There are now several commercially available molecular methods that detect antibiotic resistance genes within hours rather than days. While these methods have limitations, they offer promise for optimizing treatment and patient outcomes, and reducing further emergence of AMR. This review provides an overview of commercially available genotypic assays that detect individual resistance genes and/or resistance-associated mutations in a variety of specimen types and discusses how clinical outcomes studies may be used to demonstrate clinical utility of such diagnostics.

## Background

Antimicrobial resistance (AMR) among bacteria is an escalating public health emergency. An estimated 1.27 million deaths were attributed to bacterial AMR in 2019.^1^ If left unchecked, AMR may lead to an estimated 10 million deaths each year by 2050.^2^ Moreover, there have been dramatic global increases in AMR during the COVID-19 pandemic, largely driven by antibiotic overuse and breakdowns in infection control. It is estimated that >70% of hospitalized COVID-19 patients received antibiotics, 1 in 7 acquired a secondary bacterial infection, and up to 50% of those who died had an antibiotic-resistant infection concurrent with COVID-19.^3–6^ Advancing development and use of rapid diagnostic tests for identification of AMR is a patient care and public health priority.^7^

For patients with serious infections caused by antibiotic-resistant bacterial pathogens, prompt initiation of effective antibiotic therapy can be lifesaving. Conversely, for patients with infections caused by antibiotic-susceptible organisms, administration of narrow-spectrum agents can decrease antibiotic pressure, reduce selection for increasingly antibiotic-resistant species, and in some cases lessen toxicity, decrease cost and minimize microbiome disturbances. Thus, when making antibiotic treatment decisions, clinicians rely heavily on antibiotic susceptibility or resistance assessment; however, conventional laboratory methods may take days to identify AMR.

Most clinical microbiology laboratories use a combination of phenotypic and genotypic antimicrobial susceptibility testing (AST) strategies.^8^ Phenotypic methods evaluate microorganism growth in the presence of an antimicrobial agent. Because they rely on *in vitro* growth in culture, they are only performed after a microorganism has been recovered in pure culture, require a large inoculum and take several days for final results—time during which patients may receive suboptimal empirical antibiotic therapy. Additionally, phenotypic methods may miss pathogens present at low levels, and can be imprecise, with variable reproducibility of results. However, conventional phenotypic methods are generally less expensive than novel genotypic diagnostics (see below), provide clear information about both resistance and susceptibility, and provide an MIC value, which some clinicians use to make therapeutic decisions (Table 1). Although not discussed in this review, there are rapid phenotypic methods that determine MICs or assign susceptible, intermediate or resistant categories, such as rapid disc diffusion,^9–11^ the Pheno System (Accelerate Diagnostics)^12^ and antigen-based tests that detect protein products of resistance genes (e.g. PBP2a, encoded by *mecA*).^13^

**Table 1.** Differences between phenotypic and genotypic resistance detection methods

Characteristic	Phenotypic methods	Genotypic methods
Question to be answered	Does the antibiotic inhibit bacterial growth at clinically relevant concentrations?	Is a gene or mutation associated with antibiotic resistance present?
Turn-around time	Slow	Fast
Inoculum needed	High	Low
Provides information about resistance mechanism	No	Yes
Predicts antibiotic susceptibility and resistance	Yes	Sometimes. Only detects a gene or mutation associated with resistance; this may not correlate with phenotypic resistance in all isolates (e.g. if a gene is not expressed). If a singular genotypic resistance type is associated with resistance to a particular antibiotic in a particular bacterial species, its absence infers susceptibility. However, when there is more than one genotypic resistance type associated with resistance, such an inference may not always be correct.
Provides MIC	Yes	No
Cost	Moderate	High

In contrast, genotypic AST methods detect the presence of genes or mutations that predict AMR. These tests typically provide results within a few hours (sometimes a few minutes), and in addition to being performed on isolated bacteria, may be performed directly on certain patient specimens without requiring antecedent culturing. Such diagnostics may be configured to detect one or a small number of resistance factors, or alternatively configured as syndromic panels, which detect multiple microorganisms and resistance genes/mutations from a single specimen. Limitations of genotypic resistance detection methods are that they can detect only a subset of resistance markers for a set number of species, may overcall resistance since they detect the presence of resistance genes and/or mutations in genes, but not their expression, cannot provide MICs (at least as currently configured), and cannot detect novel resistance mechanisms. Genotypic tests also may not detect non-enzymatic resistance mechanisms like porin loss or up-regulation of efflux pumps. Additionally, genotypic resistance detection methods predict resistance, not susceptibility, and therefore cannot rule in antimicrobial therapy options unless there is a singular genetic mechanism of resistance for a particular antibiotic/bacterial species combination. They are generally more costly than older phenotypic AST methods and because they are new, do not have a track record of use in clinical practice. This raises the question of what their clinical utility might be, a question answerable with outcomes studies. Some have suggested that genotypic resistance detection assays are most useful for Gram-positive bacteria, where a single gene may reliably predict some types of resistance, but are less predictive of antibiotic susceptibility in Gram-negative bacteria, which more typically harbour a multiplicity of antibiotic resistance mechanisms (Table 1).^14^

This review provides an overview of currently available, US FDA-cleared genotypic assays that detect individual resistance genes or mutations, how they are used in clinical practice, and opportunities to advance development and uptake of these tests.

## FDA-cleared diagnostics with antibiotic resistance gene detection

A variety of FDA-cleared tests detect microorganisms as well as antibiotic resistance genes or mutations (Table 2). Tests and their clinical utility are described below.

**Table 2.** FDA-cleared tests for detection of microorganisms and antibiotic resistance genes or mutations

Specimen type	Organism	Assay and instrument (manufacturer)	Resistance genes	FDA approval
Skin and soft tissue swab	*S. aureus* (differentiates MRSA from MSSA)	Xpert MRSA/SA SSTI (Cepheid)	*mecA*, *SCCmec* and *attB* junction	2008^c^
Nasal swab	MRSA^a^	BD MAX MRSA XT (BD Diagnostics)	*mecA*, *mecC*, MREJ	2012^c^
		Xpert MRSA NxG (Cepheid)	*mecA*, junction of *SCCmec* and *attB*	2016^c^
	*S. aureus* and MRSA	Xpert MRSA/SA, MRSA Nasal Complete	*mecA*, junction of *SCCmec* and *attB*	2010^c^
		MRSA/SA ELITe MGB^®^ (ELITech Group Epoch Biosciences)	*mecA*	2012
		BD MAX STAPH SR (BD Diagnostics)	*mecA*, *mecC*, MREJ, *nuc*	2013^c^
		Cobas MRSA/SA Test (Roche)	MREJ	2014^c^
Rectal or perirectal swab or stool	VRE^b^	Xpert *vanA* (Cepheid)	*vanA*	2009^c^
	Enterobacterales, *Acinetobacter baumannii*, *P. aeruginosa*	Xpert Carba-R (Cepheid)	*bla*_IMP_, *bla*_NDM_, *bla*_VIM_, *bla*_OXA-48_, *bla*_KPC_	2018^c^
Respiratory specimens (sputum, endotracheal aspirate, BAL fluid)	Bacteria (semi-quantitative): *Acinetobacter calcoaceticus-baumannii* complex, *Klebsiella oxytoca, Serratia marcescens, Enterobacter cloacae* complex, *K. pneumoniae* group, *S. aureus*, *Escherichia coli*, *M. catarrhalis*, *Streptococcus agalactiae*, *H. influenzae*, *Proteus* species, *Streptococcus pneumoniae*, *Klebsiella aerogenes*, *P. aeruginosa*, *Streptococcus pyogenes* Other bacteria (qualitative): *Chlamydia pneumoniae*, *Legionella pneumophila*, *Mycoplasma pneumoniae* Viruses: Adenovirus, human rhinovirus/enterovirus, parainfluenza virus, coronavirus, influenza A, respiratory syncytial virus, human metapneumovirus, influenza B, MERS-CoV^c^	FilmArray PN panel (BioFire Diagnostics) ^c^FilmArray Pneumonia *plus* Panel includes detection of MERS-CoV	*mecA/C*, MREJ, *bla*_CTX-M_, *bla*_IMP_, *bla*_NDM_, *bla*_VIM_, *bla*_OXA-48-like_, *bla*_KPC_	2018^c^
Respiratory specimens (bronchoalveolar lavage-like fluids)	Bacteria: *Acinetobacter* species, *C. pneumoniae*, *Citrobacter freundii*, *E. cloacae* complex, *E. coli*, *H. influenzae*, *K. oxytoca*, *K. pneumoniae*, *Klebsiella variicola*, *L. pneumophila*, *M. catarrhalis*, *Morganella morganii*, *M. pneumoniae*, *Proteus* species, *P. aeruginosa*, *S. marcescens*, *S. aureus*, *Stenotrophomonas maltophilia*, *S. pneumoniae* Fungus: *Pneumocystis jirovecii*	Unyvero LRT BAL (Curetis GmbH)	*mecA*, *bla*_CTX-M_, *bla*_NDM_, *bla*_OXA-48_, *bla*_KPC_, *bla*_VIM_, *bla*_OXA-58_, *bla*_OXA-24_, *bla*_TEM_	2019^c^
Synovial fluid	Bacteria: *Anaerococcus prevotii/vaginalis*, *Bacteroides fragilis*, *Citrobacter*, *Clostridium perfringens*, *Cutibacterium avidum/granulosum*, *E. cloacae* complex*, Enterococcus faecalis*, *Enterococcus faecium*, *E. coli*, *Finegoldia magna*, *H. influenzae*, *K. kingae*, *K. aerogenes*, *K. pneumoniae* group, *M. morganii*, *Neisseria gonorrhoeae*, *Parvimonas micra*, *Peptoniphilus*, *Peptostreptococcus anaerobius*, *Proteus* species, *P. aeruginosa*, *Salmonella* species, *S. marcescens*, *S. aureus*, *Staphylococcus lugdunensis*, *Streptococcus* species, *S. agalactiae*, *S. pneumoniae*, *S. pyogenes* Fungi: *Candida* species, *Candida albicans*	JI panel (BioFire Diagnostics)	*mecA/C*, *vanA/B*, MREJ, *bla*_CTX-M_, *bla*_IMP_, *bla*_NDM_, *bla*_VIM_, *bla*_OXA-48-like_, *bla*_KPC_	2022^c^
Blood, multiple organism types	Bacteria: *E. faecalis*, *E. faecium*, *Listeria monocytogenes*, *Staphylococcus* species, *S. aureus*, *S. epidermidis*, *S. lugdunensis*, *Streptococcus* species, *S. agalactiae*, *S. pneumoniae*, *S. pyogenes* *A. calcoaceticus-baumannii* complex, *B. fragilis*, *H. influenzae*, *Neisseria meningitidis*, *P. aeruginosa*, *S. maltophilia*, Enterobacterales, *E. cloacae* complex, *E. coli*, *K. aerogenes*, *K. oxytoca*, *K. pneumoniae* group, *Proteus* species, *Salmonella* species, *S. marcescens* Fungi: *C. albicans*, *Candida auris*, *Nakaseomyces glabrata (Candida glabrata)*, *Pichia kudriavzevii (Candida krusei)*, *Candida parapsilosis*, *Candida tropicalis*, *Cryptococcus neoformans/gattii*	Blood Culture Identification Panel (BCID) (BioFire Diagnostics)	BCID: *mecA*, *vanA/B*, *bla*_KPC_ BCID2: *bla*_CTX-M_, *bla*_IMP_, *bla*_NDM_, *bla*_VIM_, *bla*_OXA-48-like_, *bla*_KPC_*mcr-1*, *mecA/C*, MREJ, *vanA/B*	BCID in 2013^c^; BCID2 in 202°^c^
Blood, Gram-positive species	*S. aureus* (differentiates MRSA from MSSA)	Xpert MRSA/SA Bc (Cepheid)	*mecA*, *SCCmec* insertion at a*ttB*	2008^c^
	*S. aureus*, *S. epidermidis*	Verigene Staphylococcus Blood Culture (Bc-S) Nucleic Acid Test (Luminex)	*mecA*	2011^c^
	*Staphylococcus* species, *S. aureus*, *S. epidermidis*, *S. lugdunensis*, *E. faecalis*, *E. faecium*, *Streptococcus* species, *S. pneumoniae*, *S. pyogenes*, *S. agalactiae*, *Streptococcus anginosus* group, *Listeria* species	Verigene Bc-GP (Luminex)	*mecA*, *vanA*, *vanB*	2012^c^
	*S. aureus*, *S. lugdunensis*, *Staphylococcus* species	Staph ID/R Blood culture panel (Great Basin)	*mecA*	2016^c^
	*S. aureus*, *S. epidermidis*, *S. pneumoniae*, *E. faecalis*, *E. faecium*	iC-GPC (iCubate)	*mecA*, *vanA/B*	2017^c^
	*Bacillus cereus group*, *Bacillus subtilis* group, *Corynebacterium* species, *C. acnes*, *Enterococcus* species, *E. faecalis*, *E. faecium*, *Lactobacillus* species, *Listeria* species, *L. monocytogenes*, *Micrococcus* species, *Staphylococcus* species, *S. aureus*, *S. epidermidis*, *S. lugdunensis*, *Streptococcus* species, *S. agalactiae*, *S. anginosus* group, *S. pneumoniae*, *S. pyogenes*	ePlex BCID-GP (GenMark Diagnostics)	*mecA*, *mecC*, *vanA*, *vanB*	2018^c^
Blood, Gram-negative species	*Acinetobacter* species, *Citrobacter* species, *Enterobacter* species, *Proteus* species, *E. coli*, *K. pneumoniae*, *K. oxytoca*, *P. aeruginosa*	Verigene Bc-GN (Luminex)	*bla*_CTX-M_, *bla*_IMP_, *bla*_NDM_, *bla*_VIM_, *bla*_OXA_, *bla*_KPC_	2014^c^
	*A. baumannii* complex, *E. cloacae* complex, *E. coli*, *K. oxytoca*, *K. pneumoniae*, *P. aeruginosa*, *Proteus* species, *S. marcescens*	iC-GN (iCubate)	*bla*_KPC_, *bla*_NDM_, *bla*_CTX-M-group_	2019^c^
	*A. baumannii*, *B. fragilis*, *Citrobacter*, *Cronobacter sakazakii*, *Enterobacter (non-cloacae* complex), *E. cloacae* complex, *E. coli*, *Fusobacterium nucleatum*, *Fusobacterium necrophorum*, *H. influenzae*, *K. oxytoca*, *K. pneumoniae* group, *M. morganii*, *N. meningitidis*, *Proteus* species, *Proteus mirabilis*, *P. aeruginosa*, *Salmonella*, *Serratia* species, *S. marcescens*, *S. maltophilia*	ePlex BCID-GN (GenMark Diagnostics)	*bla*_CTX-M_, *bla*_IMP_, *bla*_NDM_, *bla*_VIM_, *bla*_OXA-48-like,_*bla*_KPC_	2019^c^
Bacterial colonies	Enterobacterales, *A. baumannii*, *P. aeruginosa*	Xpert CARBA-R (Cepheid)	*bla*_IMP_, *bla*_NDM_, *bla*_VIM_, *bla*_OXA-48_, *bla*_KPC_	2018^c^
		GenePOC CARBA (GenePOC Inc.)	*bla*_IMP_, *bla*_NDM_, *bla*_VIM_, *bla*_OXA-48-like_, *bla*_KPC_	2019^c^
	Enterobacterales, *P. aeruginosa*, *E. faecalis*	Acuitas AMR Gene Panel (OpGen, Inc.)	*aac*, *aad*, *ant*, *aph*, *armA*, *bla*_CMY_, *bla*_CTX-M-1_, *bla*_CTX-M-2_, *bla*_CTX-M-9_, *dfr*, *dha*, *bla*_IMP_, *bla*_KPC_, *mcr-1*, *bla*_NDM_, *bla*_OXA-1_, *bla*_OXA-48_, *bla*_OXA-9_, *per*, *rmt*, *shv, sul1*, *sul2*, *bla*_TEM_, *vanA*, *veb*, *bla*_VIM_, *P. aeruginosa gyrA* mutant, *E. coli gyrA* mutant	2021

FDA-cleared tests are listed at https://www.fda.gov/medical-devices/in-vitro-diagnostics/nucleic-acid-based-tests#microbial.

a FDA-cleared tests for MRSA detection that are no longer available in the US: IDI-MRSA assay (Infectio Diagnostic Inc.), BD GeneOhm MRSA ACP (BD Diagnostics), NucliSENS EasyQ^®^ MRSA Assay (bioMérieux), mecA Express FISH (AdvanDx Inc.).

b FDA-cleared tests for VRE detection that are no longer available in the US: BD GeneOhm VanR assay (BD Diagnostics), IMDx vanR for Abbott m2000 (Intelligent Medical Devices, Inc.).

c Conformite Europeenne (Ce) marked.

### Lower respiratory tract specimens

The BioFire FilmArray Pneumonia (PN) Panel received FDA clearance in 2018 for testing of respiratory specimens. It uses nested multiplex PCRs to detect 33 targets, including traditionally culturable bacteria, bacteria that do not grow in routine cultures, and viruses, alongside seven resistance genes encoding methicillin resistance [*mec*A/C and SCC*mec* right extremity junction (MREJ)], ESBLs (*bla*_CTX-M_) and carbapenemases (*bla*_IMP_, *bla*_KPC_, *bla*_NDM_, *bla*_OXA-48-like_ and *bla*_VIM_). Resistance genes are reported as detected only if a plausible microorganism that could harbour the gene is present at >10^3.5^ copies/ml. The FilmArray Pneumonia *plus* Panel includes MERS-CoV and is also FDA-cleared.^15^ To potentially help distinguish between colonization or infection with some species, the test provides semi-quantitative results for a subset of 15 bacterial targets recoverable in traditional cultures. The turn-around time is approximately 1 h.

Despite its approval several years ago, the PN panel has not been widely implemented in the USA. Some clinicians and microbiologists have raised concerned that molecular detection of organisms from non-sterile sources may lead to detection of colonizing bacteria and hence overtreatment with antibiotics. Additionally, whether semi-quantitative results accurately distinguish colonization from infection is unclear. Detection of a resistance gene cannot be definitively linked to the microorganism(s) detected. The PN panel does not detect emerging ESBL or carbapenemase genes.

In studies evaluating performance of the PN panel, sensitivity varied by target pathogen and respiratory specimen type.^16^ While some studies demonstrated >90% sensitivity for all bacterial targets, one study demonstrated lower sensitivity (75%) for *Enterobacter aerogenes* in bronchoalveolar lavage (BAL) specimens.^15^ The sensitivity of resistance gene detection in sputum and BAL was lower for *bla*_CTX-M_ (80%–85%) than for other genes. Specificity was >90% for almost all targets.^15,17,18^ Another study found a positive percent agreement (PPA) of ∼95% between the PN panel and culture and PCR methods, except for *Pseudomonas aeruginosa* (PPA of 75%) and *Staphylococcus aureus* (PPA 89%).^19^ Similar test performance, including a few false-negative results for *P. aeruginosa* and *S. aureus* was found in an evaluation of 259 BAL specimens from inpatients at 8 US hospitals.^20^ In a multinational evaluation of over 1200 specimens each of BAL and sputum, the Pneumonia *plus* Panel was compared with standard-of-care methods and had a PPA of 93% and a negative percent agreement (NPA) of 96% for detection of common bacteria.^21^

Whether its use can lead to more effective or judicious antibiotic use or improved patient outcomes has not been well studied. Potential impact on prescribing has been evaluated in a few studies, which suggest that use of the PN panel could have resulted in less inappropriate antibiotic therapy or faster antibiotic de-escalation in half to two-thirds of patients.^19,20^

The Curetis Unyvero (Curetis GmbH) was FDA-cleared in 2018 for testing of endotracheal aspirates or BAL-like specimens in hospitalized adults. It targets 19 bacteria and 10 antibiotic resistance genes and a turn-around time of ∼5 h.^16^ It does not provide target quantitation. In a multicentre study including 603 specimens, sensitivity for organism identification compared with culture was 93%, with PPA ranging from 88% to 100%. The PPA range for resistance gene detection was 80%–100% for both sputum and BAL specimens.^22^ In a larger study of nearly 1400 BAL specimens collected from 11 sites in the USA, PPA was 93% and NPA was 98% for bacterial identification, compared with culture.^23^

### Synovial fluid specimens

The BioFire Joint Infection (JI) Panel received FDA clearance in 2022. It is performed on a synovial fluid and detects 31 bacteria or yeast, and 11 resistance determinants (*vanA/B*, *mecA/C* and MREJ, *bla*_CTX-M_, *bla*_IMP_, *bla*_VIM_, *bla*_NDM_, *bla*_KPC_, *bla*_OXA-48-like_) in ∼1 h.

Test performance has been evaluated in a few studies. In a study of 45 consecutive synovial fluid specimens collected from two US institutions, results from the JI panel were compared with conventional culture of synovial fluid. The JI panel had a specificity of 98% and negative predictive value of 100%.^24^ In a pilot evaluation in four US and French hospitals, synovial fluid from 235 patients with suspected osteoarticular infections was tested using the JI panel and conventional methods. More positive specimens were detected using the JI panel than culture (77 versus 55); among specimens with positive cultures, 78% had concordant organisms detected by the JI panel.^25^ In the largest study to date, across nine hospitals in the USA and Europe, 925 synovial fluids were evaluated using the panel and conventional methods.^26^ The panel detected more positive specimens compared with culture (145 versus 124) and 95% of positive results were concordant with culture or comparator PCR results.^26^ A recent study showed that sensitivity of the JI panel for periprosthetic joint infection diagnosis was low, largely due to the absence of *Staphylococcus epidermidis* on the panel.^27^ The JI panel also does not include *Cutibacterium acnes*, an important cause of periprosthetic joint infection, which may limit its utility. There are no studies evaluating clinical outcomes with use of the JI panel. The JI panel may add value in cases of antibiotic pretreatment when cultures may be negative, but this requires further study.

### Positive blood culture bottles

There are several FDA-cleared platforms for molecular detection of bacteria and select antibiotic resistance genes from positive blood culture bottles. No test is currently available for direct detection of genotypic antibiotic resistance directly from blood. Some available commercial platforms for testing positive blood culture bottles, including the BioFire and ePlex assays, are multiplex PCR assays that enable detection of a variety of bacteria and fungi as well as select antibiotic resistance genes. The Verigene system is based on bacterial DNA hybridization with target microarrays rather than PCR amplification. For both the Verigene and ePlex assays, different panels are used for blood cultures that have Gram-positive versus Gram-negative bacteria visualized on Gram stain. These platforms detect ∼80% of organisms that cause bacteraemia in the USA.^28,29^ Other cleared genotypic AMR tests for positive blood cultures detect fewer pathogens, either multiple Gram-positive targets, or solely *S. aureus* and methicillin resistance. These methods allow target detection within one or a few hours and are easy to use but more expensive than conventional culture and susceptibility testing. Test performance is good in monomicrobial cultures, with sensitivity and specificity for most targets >95%.^30–34^ In polymicrobial blood cultures, sensitivity for detecting all organisms is lower (54%–71%), a known limitation of molecular blood culture diagnostics.^30,35^ In a recent survey of US clinical microbiology laboratories, 90/96 (94%) reported using at least one rapid blood culture method, most commonly the BioFire FilmArray.^36^

Unlike for other specimen types, there have been several studies evaluating clinical outcomes using molecular blood culture diagnostics. Clinical impact depends upon multiple institution-specific factors, including local pathogen resistance rates, patient populations, antimicrobial prescribing practices, and antimicrobial stewardship programme activities. Clinical impact also varies by study design. Several retrospective pre–post observational studies using historical control groups, and a small number of randomized controlled trials have been performed. The overwhelming majority demonstrate decreased time to optimal therapy with rapid diagnostics. While some observational studies have reported rapid diagnostics to be associated with decreased lengths of stay, lower mortality and reduced costs,^31,37–39^ others, including two recent randomized controlled trials,^28,29^ have shown no differences in mortality and length of stay compared with conventional subculture and susceptibility testing. In US-based trials, pairing molecular blood culture diagnostics with antibiotic stewardship interventions enables faster antibiotic de-escalation compared with conventional culture and susceptibility testing.^29^

### Skin swabs

One FDA-cleared method detects colonization or infection with MRSA and MSSA from skin and skin structure swabs. The Cepheid MRSA/SA SSTI test is a real-time PCR performed on swabs from skin wounds without requiring bacterial growth in culture. In under 1 h, it detects *S. aureus* (*spa* gene), *mecA*, the gene that confers methicillin resistance, and the chromosomal insertion site, SCC*mec*. It was cleared in 2008 and has a PPA of 94% and NPA of 97% compared with culture.^40^ Test performance is slightly lower in subjects with prior antibiotic use. A recent pre–post study in Spain found that compared with a historical control cohort, use of the MRSA/SA SSTI test in a prospective cohort of 155 hospitalized adult patients with skin infections, including surgical site infections, diabetic foot wounds, abscesses and cellulitis, paired with advice from an antibiotic stewardship programme, led to improvements in mortality, length of stay, and days of therapy.^41^ This test has also been used to test bone and joint specimens and BAL fluid but is not approved by the FDA for testing these specimens.^42–44^

### Nasal swabs

There are several commercially available tests that allow rapid detection of MRSA colonization from nasal swabs. All detect the methicillin resistance gene *mecA* and some also detect *mecC* and/or the insertion of *SCCmec* into *attB* or *orfX.* The Xpert SA Nasal Complete detects *mecA/C*, *spa* and *SCCmec-orfX* from nasal swabs in under 3 h. It has a sensitivity ranging from 92% to 100% and a specificity of 98%–100%, although sensitivity may be lower in low-resource settings.^45–47^ Tests from other manufacturers have comparable performance.^42^

MRSA nasal screening is used for several indications. Admission screening for MRSA has been done in US Veterans Affairs (VA) hospitals, and many neonatal ICUs,^48^ enabling prompt placement of colonized patients on contact isolation. MRSA nasal screening is also often used to rule out MRSA infection in the respiratory tract and other sites, with a negative predictive value (NPV) of 97%–99% making it an effective antimicrobial stewardship tool to withhold or reduce unnecessary anti-MRSA therapy.^49–52^ Recent US guidelines for management of community-acquired pneumonia in adults recommend that a negative rapid nasal PCR for MRSA can be used to withhold anti-MRSA treatment for non-severe pneumonia.^53^

Nasal screening for MRSA has been used for pre-operative screening of colonization prior to prosthetic joint, spine and cardiac surgery. A positive MRSA screen may lead to pre-operative decolonization with mupirocin and/or anti-MRSA perioperative antibiotic prophylaxis. Both British and US surgical society guidelines recommend that MRSA-colonized patients who require surgery receive perioperative prophylaxis that includes a glycopeptide antibiotic.^54–56^

MRSA nasal colonization tests are also relied upon for hospital infection control activities. For over a decade, the US CDC has recommended that MRSA-colonized patients be placed on contact precautions and cohorted during outbreaks,^57,58^ although recent data suggest that widespread use of contact precautions for MRSA does not impact in-hospital transmission and should be reconsidered.^59^

### Rectal swabs

There are two PCR tests for detection of rectal carriage of VRE or carbapenemase-producing Enterobacterales (CP-CRE). The Xpert assay detects *vanA* in enterococci with 98% PPA and 92% NPV compared with culture.^60^ The Xpert Carba-R detects five carbapenemase genes (*bla*_IMP_, *bla*_VIM_, *bla*_KPC_, *bla*_NDM_ and *bla*_OXA-48-like_). Testing can be performed on rectal or perirectal swabs, stool or colonies. Compared with reference culture and sequencing, the Xpert Carba-R test had a sensitivity of 97%, specificity of 99%, positive predictive value (PPV) of 95%, and NPV of 99%.^61^ Many Gram-negative species have other carbapenem resistance mechanisms that will not be detected using this assay.

To reduce healthcare-associated infections, facilities may consider screening individuals for colonization with VRE or CP-CRE. Such selective screening may be performed for high-risk hosts, including but not limited to those who are immunocompromised, undergoing dialysis, have chronic underlying conditions and frequent hospitalizations,^57,58^ or are contacts of infected patients. Additionally, active surveillance may be performed on admission or on patients meeting pre-specified criteria.^62^

## Implementation considerations

The clinical impact of rapid diagnostics depends not just on test performance, but also on how tests are implemented. Institutions must decide whether to offer molecular diagnostics for AMR for all or a subset of patients, and whether to perform testing 24/7 or at limited times. Because genotypic resistance detection methods may occasionally provide discordant results when compared with phenotypic susceptibility results, clinical laboratories should have strategies in place for addressing such discordant results. Clinical laboratories should implement a workflow for resolving phenotypic/genotypic resistance discrepancies, from checking purity plates to rule out polymicrobial cultures, investigating lot numbers and reagents for contamination or expiration, and resolving clerical errors in reporting, to name a few possibilities.^63^ The CLSI has recommendations for discrepancy resolution.^64^

Implementation of certain rapid genotypic diagnostic assays together with oversight of an antibiotic stewardship team results in faster, more appropriate antibiotic therapy decisions by clinicians, as demonstrated in a randomized clinical trial of testing of positive blood culture bottles.^29^ Stewardship teams can assist with test interpretation and encourage clinicians to act promptly on critical results, which is especially important for new diagnostics with which clinicians may be unfamiliar.

Additionally, to maximize clinical impact, tests that detect the presence of resistance genes should be reported with interpretation and therapeutic guidance where possible (Table 3).^36^ There is variability in how laboratories report results of AMR markers and organisms.^36^ Additional challenges in providing timely rapid diagnostic test results occur in centres where specimens must be transported to off-site microbiology laboratories.

**Table 3.** Reporting comments for molecular blood culture diagnostics. Adapted from Banerjee *et al*.^29^

*Staphylococcus aureus*, *mecA* detected	Probable methicillin-resistant *Staphylococcus aureus* (MRSA); further testing in progress. MRSA is predictably resistant to beta-lactam antibiotics (except ceftaroline). Patient requires contact precautions if hospitalized.
*Staphylococcus aureus*, *mecA* not detected	Methicillin (oxacillin)-susceptible *Staphylococcus aureus*. Preferred therapy is an anti-staphylococcal β-lactam antibiotic, unless clinically contraindicated.
*Staphylococcus*, coagulase-negative, *mecA* detected	Methicillin (oxacillin)-resistant coagulase-negative *Staphylococcus*. Possible blood culture contaminant (unless isolated from more than one blood culture draw or clinical case suggests pathogenicity). No antibiotic treatment is indicated for blood culture contaminants.
*Enterobacter cloacae* complex	This organism may contain an inducible β-lactamase. Penicillin or second- or third-generation cephalosporin monotherapy may result in emergence of high-level resistance.
*Escherichia coli*, *bla*_CTX-M_ detected	Extended-spectrum β-lactamase-producer. Carbapenems are drugs of choice for ESBL-producers.
*E. coli*, *bla*_KPC_ detected	Carbapenemase producer. Patient requires contact precautions if hospitalized. This organism is resistant to carbapenems and other β-lactam antibiotics. Consult infectious diseases.

## Future opportunities for test development and increased uptake

### Faster and quantitative AMR detection methods

Despite tremendous advances in infectious disease diagnostics, unmet diagnostic needs remain. Development of novel genotypic AMR detection platforms that can be performed directly on clinical specimens rather than organisms growing in culture speed up identification of resistant infections by a day or more and simplify laboratory workflows. A panel that offers direct-from-blood organism detection is FDA-cleared; the T2 Resistance Panel, a similar, novel panel that detects AMR genes directly from blood specimens is in development (T2 Biosystems).^65^ Diagnostics that detect organisms and resistance genes directly from genital or urine specimens are also available or in development.^66,67^ Point-of-care assays that can be completed during a clinical encounter may support real-time treatment decisions and prompt infection control interventions. Shortening workflows and cost to make WGS of pathogens feasible to incorporate into routine clinical care would also enable rapid detection of AMR.

Future studies should clarify how to interpret rapid PN panels and quantitation of detected organisms. It is especially difficult to interpret detection of organisms that can be either airway colonizers or pathogens, like *Streptococcus pneumoniae*, *Haemophilus influenzae* and *Moraxella catarrhalis*. Test performance should be evaluated in populations with high rates of airway colonization, such as children and chronically ventilated or tracheostomy-dependent patients and patients with cystic fibrosis.

Diagnostics that link species with resistance genes would aid clinical decision-making and appropriate antibiotic use, especially for polymicrobial specimens. Because current diagnostics cannot universally definitively determine which microorganism harbours a resistance gene, clinicians may prescribe antibiotics that are broader than necessary. For example, when a blood culture diagnostic detects the presence of *S. aureus*, *S. epidermidis* and *mecA* in a specimen, clinicians may decide to treat MRSA, even though the methicillin resistance may be associated with S. *epidermidis*, a likely skin contaminant. Similarly, when a pneumonia diagnostic detects *Klebsiella pneumoniae*, *P. aeruginosa* and a carbapenemase gene from a respiratory specimen, clinicians may opt to treat a potential CRE pneumonia, even if the true pathogen is a carbapenem-susceptible *P. aeruginosa*.

### Robust clinical utility studies that demonstrate value of genotypic resistance detection methods

Unclear clinical utility of rapid genotypic resistance detection limits test uptake and may result in unfavourable reimbursement decisions by payers. Clinicians, hospital leaders and payers may be convinced about the value of rapid molecular genotypic resistance detection through clinical utility studies that include an array of endpoints, including but not limited to laboratory efficiencies, infection control and antibiotic stewardship metrics, and patient, institutional and societal outcomes (Table 4). Benefits on mortality and length of stay are not necessarily expected as these are multifactorial outcomes that can be impacted by comorbid conditions other than infection. Studies evaluating mortality and length of stay as primary outcomes may require large sample sizes and careful consideration of inclusion criteria, and even then, a mortality benefit may not be shown. A mortality benefit of timelier effective antibiotic treatment would likely best be demonstrated in a population where AMR is common, and the rapid diagnostic result would often lead to antibiotic escalation for subjects receiving ineffective empirical antibiotic treatment. In the end, facilitation of de-escalation or sparing of antibiotics should be sufficient to justify implementation of a novel diagnostic.

**Table 4.** Possible endpoints for clinical outcomes studies evaluating rapid diagnostics with antibiotic resistance gene or mutation detection

Laboratory outcomes	Laboratory workflow efficiency
	Test performance compared to historical standard
	Technologist satisfaction
	Cost
Infection control outcomes	Time to isolation
	Isolation room turnover
	Acquisition of antibiotic-resistant organisms
	Spread of antibiotic-resistant organisms
Antimicrobial stewardship outcomes	Time to effective or appropriate antimicrobial therapy
	Time to appropriate antibiotic de-escalation
	Number of stewardship recommendations
	Acceptance rate of stewardship recommendations
	Acquisition of antibiotic-resistant organisms
Patient outcomes	Mortality
	Length of hospitalization
	Adverse events
	Patient satisfaction
	Lack of clinical response
	Readmission
	Additional laboratory testing, imaging, procedures, or surgery
	Cost
Institutional outcomes	Length of hospitalization
	Additional laboratory testing, imaging, procedures, or surgery
	Cost
	Spread of antibiotic-resistant organisms
	Emergency department triage time
Societal outcomes	Cost
	Spread of antibiotic-resistant organisms

Clinical utility studies should incorporate endpoints that are downstream effects of improved clinical decision-making, such as workflow and efficiency benefits for the laboratory, decreased time and expertise needed for testing, faster turnover of isolation rooms and triaging in the Emergency Department, timely identification of patients requiring isolation or cohorting, and avoidance of hospitalization. There are also likely to be population-level (societal) reductions in AMR through more judicious antibiotic use, although these benefits may not be seen immediately and are challenging to ascertain in a single study. Endpoints may differ by type of tests and population, including inpatient, outpatient and special populations (e.g. children, immunocompromised hosts). Cost-effectiveness evaluations of molecular AMR detection assays should consider not just the cost of the test, but also the savings resulting from potential downstream benefits. Such evaluations will require broader-value frameworks that capture long-term clinical and economic benefits to a health system, as previously reviewed.^68^

Once clinical outcomes studies demonstrate that molecular AMR diagnostics add value, future clinical guidelines should incorporate such diagnostics into recommended testing algorithms. This can guide payers in supporting reimbursement of such tests and hospital administrators in appreciating the need to invest in them.

### Making genotypic resistance testing accessible and affordable

The cost of genotypic AMR diagnostics is often high, which can be challenging in low- and middle-income (LMIC) settings, where AMR rates are highest. Global collaboration and public/private partnerships have made diagnostics for other pathogens accessible and affordable; such strategies should be considered for AMR diagnostics. For example, the Xpert MTB/RIF assay, which simultaneously detects *Mycobacterium tuberculosis* complex DNA and rifampicin resistance (a reliable surrogate for MDR-TB) was developed within 4 years through a collaboration among academic and industry partners.^69–71^ These groups pooled resources needed for test development, validation and field evaluations, and industry partners agreed to flexible product pricing,^72^ all of which led to endorsement by the WHO.^73^ Similarly, the Access to COVID-19 Tools (ACT) Accelerator was launched in 2020 as a global collaboration between scientists, businesses, philanthropists and global health organizations to develop and distribute tests, treatments and vaccines for COVID-19,^74^ and reduced the cost of COVID-19 tests to less than $3 USD for LMICs. Similar policy interventions are needed to increase broad uptake of genotypic AMR diagnostics, especially in LMICs, where the need is greatest.

## Conclusions

Advancing development and use of rapid diagnostic tests for identification of AMR is a public health and patient care priority. There are currently multiple FDA-cleared genotypic assays that detect individual resistance genes and some mutations from a variety of specimen types. These assays offer hope that rapid resistance detection can lead to more judicious use of antibiotics and reduce emergence and spread of AMR. Robust outcomes studies that demonstrate value of these tests and policies to make them available are needed.

## Transparency declarations

R.B. has none to declare. R.P. reports grants from ContraFect, TenNor Therapeutics Limited and BIOFIRE. R.P. is a consultant to PhAST, Torus Biosystems, Day Zero Diagnostics, Mammoth Biosciences and HealthTrackRx; monies are paid to Mayo Clinic. Mayo Clinic and R.P. have a relationship with Pathogenomix. R.P. has research supported by Adaptive Phage Therapeutics. Mayo Clinic has a royalty-bearing know-how agreement and equity in Adaptive Phage Therapeutics. R.P. is also a consultant to Netflix, Abbott Laboratories, Oxford Nanopore Technologies and CARB-X. In addition, R.P. has a patent on *Bordetella pertussis/parapertussis* PCR issued, a patent on a device/method for sonication, with royalties paid by Samsung to Mayo Clinic, and a patent on an anti-biofilm substance issued. R.P. receives honoraria from the NBME, Up-to-Date and the Infectious Diseases Board Review Course.
